# Identification of α-Chimaerin as a Candidate Gene for Critical Period Neuronal Plasticity in Cat and Mouse Visual Cortex

**DOI:** 10.1186/1471-2202-12-70

**Published:** 2011-07-18

**Authors:** Cui Bo Yang, Yu Ting Zheng, Paul J Kiser, George D Mower

**Affiliations:** 1 Department of Anatomical Sciences and Neurobiology, University of Louisville School of Medicine, 500 S. Preston St., Louisville, KY 40202, USA; 2 Department of Medicine, University of Louisville School of Medicine, 500 S. Preston St., Louisville, KY 40202, USA; 3 Department of Biology, Bellarmine University, 2001 Newburg Rd., Louisville, KY 40205, USA

**Keywords:** Chimerin, α1-Chimaerin, α2-Chimaerin, Dark Rearing, differential display PCR

## Abstract

**Background:**

In cat visual cortex, critical period neuronal plasticity is minimal until approximately 3 postnatal weeks, peaks at 5 weeks, gradually declines to low levels at 20 weeks, and disappears by 1 year of age. Dark rearing slows the entire time course of this critical period, such that at 5 weeks of age, normal cats are more plastic than dark reared cats, whereas at 20 weeks, dark reared cats are more plastic. Thus, a stringent criterion for identifying genes that are important for plasticity in visual cortex is that they show differences in expression between normal and dark reared that are of opposite direction in young versus older animals.

**Results:**

The present study reports the identification by differential display PCR of a novel gene, α-chimaerin, as a candidate visual cortex critical period plasticity gene that showed bidirectional regulation of expression due to age and dark rearing. Northern blotting confirmed the bidirectional expression and 5'RACE sequencing identified the gene. There are two alternatively-spliced α-chimaerin isoforms: α1 and α2. Western blotting extended the evidence for bidirectional regulation of visual cortex α-chimaerin isoform expression to protein in cats and mice. α1- and α2-Chimaerin were elevated in dark reared compared to normal visual cortex at the peak of the normal critical period and in normal compared to dark reared visual cortex at the nadir of the normal critical period. Analysis of variance showed a significant interaction in both cats and mice for both α-chimaerin isoforms, indicating that the effect of dark rearing depended on age. This differential expression was not found in frontal cortex.

**Conclusions:**

Chimaerins are RhoGTPase-activating proteins that are EphA4 effectors and have been implicated in a number of processes including growth cone collapse, axon guidance, dendritic spine development and the formation of corticospinal motor circuits. The present results identify α-chimaerin as a candidate molecule for a role in the postnatal critical period of visual cortical plasticity.

## Background

The postnatal development of visual cortex is guided by visual experience during early postnatal life. The clearest example of such environmental effects on visual cortical development is monocular deprivation, a condition that leads to dramatic anatomical and physiological abnormalities [1]. In normal development, sensitivity to monocular deprivation is limited to a "critical period," which in cats, begins several weeks after birth, peaks at about 5-6 weeks, gradually declines to low levels at 5 months and disappears at about 1 year of age [1]. Mice show a similar critical period for monocular deprivation but with a shorter time course [2].

Rearing in total darkness from birth maintains many properties of the neonatal visual cortex in cats [3-5] and mice [2,6]. Thus, visual cortical neurons of dark reared animals show reduced responsiveness, enhanced response habituation and reduced selectivity for the orientation and direction of movement of a visual stimulus compared to age matched normally reared animals. When subsequently exposed to the visual environment, normal neuronal response properties emerge and more importantly, dark rearing also extends the critical period for effects of monocular deprivation far beyond its normal age limit in both cats [3,4] and mice [6]. Electrophysiological results indicate that the effect of dark rearing is to slow the time course of the entire critical period. At young ages (5 weeks) normal animals are more plastic than dark reared, while at later ages (20 weeks) dark reared animals are more plastic [7,8]. Thus, a stringent criterion for identifying genes that control plasticity in visual cortex is that they show opposite direction differences in their levels of expression between normal and dark rearing in young versus older animals.

We have completed a differential display PCR (ddPCR) screen of visual cortex of normal and dark reared cats at 5 and 20 weeks to identify such candidate plasticity genes. Northern and western blots were used to verify opposite direction effects of dark rearing in young versus older cats and mice. We have repeatedly found two patterns of differential expression in cat and mouse visual cortex. One pattern (eg. Dab-1) [9] is an elevation in normal animals at the peak of the critical period (cats: 5 weeks; mice: 3.5 weeks) and an elevation in dark reared animals at the nadir of the normal critical period (cats: 20 weeks; mice: 9.5 weeks). This pattern could represent genes that activate plasticity mechanisms. The other pattern is the opposite: elevation in dark reared animals at the peak of the normal critical period and elevation in normal animals at its nadir (eg. Munc13-3) [10,11] and could represent plasticity repressor genes.

Here we report the identification of another novel gene, α-chimaerin, as a candidate visual cortex critical period plasticity gene. There are two alternatively-spliced α-chimaerin transcript variants, α1 and α2. Both isoforms are expressed in postnatal and adult brain but only α2 in embryonic brain [12]. Chimaerins have been implicated in axonal guidance and NMDA dependent dendritic spine regulation [13-18]. Both isoforms showed elevated expression in dark reared animals at the peak of the normal critical period and elevation in normal animals at the nadir of the critical period.

## Results

### Identification of α-Chimaerin as a Candidate Plasticity Gene in Cat Visual Cortex

**Figure 1:**
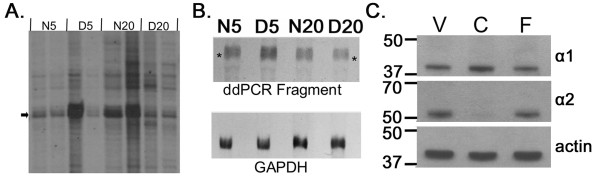
**Differential expression of α-chimaerin: Identification by ddPCR, confirmation by northern blot and validation of antibodies**.  A. Portion of a ddPCR sequencing gel showing a ddPCR product (arrow) which is expressed more highly in dark reared (D5) than normal (N5) cat visual cortex at 5 weeks and more highly in normal (N20) than dark reared (D20) cat visual cortex at 20 weeks. Independently isolated RNA samples from two cats at each rearing/age condition were run together (8 lanes) to help eliminate false positives. B. Top. Northern blot confirming differential expression of the ddPCR fragment. Total cat visual cortical RNA from each of the four rearing/age conditions was loaded and probed with the cloned ddPCR fragment. Asterisks indicate 28s rRNA. Bottom. The same filter is shown after stripping and reprobing with GAPDH. C. Western blots indicated that each antibody recognized a single band of appropriate molecular weight (α1: 38 kD, α2: 53 kD). α1-Chimaerin (Top) was expressed about equally in mouse visual (V), frontal (F) cortex and cerebellum (C). α2-Chimaerin (Middle) was expressed highly in neocortical structures but at very low levels in cerebellum. Filter was stripped and reprobed with an antibody to actin (Bottom) as a control for loading errors.

Figure 1A shows ddPCR results from a primer pair that revealed a band that showed elevated expression in dark reared cat visual cortex at 5 weeks and in normal cat visual cortex at 20 weeks. There was likely a loading error on the right D5 sample. The band was excised and cloned to generate probes for northern blotting and for cloning and sequencing. Northern blotting with the ddPCR fragment confirmed bidirectional regulation due to age and dark rearing (Figure 1B) and densitometric analysis, corrected against GAPDH, indicated a 1.9 fold increase in expression in D5 compared to N5 and a 1.7 fold increase in N20 compared to D20 visual cortex.

Sequencing was done to establish the identity of the bidirectionally regulated gene. The 153 bp ddPCR fragment showed 91% identity with the 3' end of human α-chimaerin. 5' RACE was done to extend the sequence to 2379 bases of the cat gene (GenBank: EU661869.2) which included the complete coding region. Blast search of the coding region indicated 98% identity to the human chimaerin gene on chromosome 2.

### Bidirectional Regulation of α-Chimaerin Protein in Cat and Mouse Visual Cortex

To extend the mRNA results to protein, western blots in cat and mouse visual cortex were done. As shown in figure 1C, the antibodies developed as described in Materials and Methods to the two isoforms of α-chimaerin (α1 and α2) each labeled a single band of appropriate size (α1: 38 kD, α2: 53 kD). α1-Chimaerin was expressed in visual cortex, frontal cortex and cerebellum. α2-Chimaerin was expressed in the two neocortical structures but not detected in cerebellum. These regional differences in expression between the two protein isoforms match the regional differences in their mRNA expression [12] further confirming the specificity of the antibodies.

**Figure 2:**
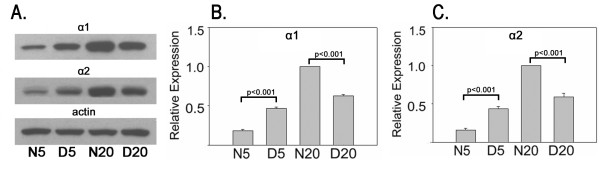
**Bidirectional regulation of α-chimaerin protein expression by age and dark rearing in cat visual cortex. ** A. Western blots showing levels of α1- and α2-chimaerin and actin protein expression in cat visual cortex. Same blot was stripped and reprobed with the three antibodies. B and C. Densitometric results for α1- and α2-chimaerin protein levels in cat visual cortex. Mean +/- S.E. of protein expression as determined by densitometry (corrected against actin to correct for loading errors) from three independent groups of cats are plotted. Data from each group were normalized against N20 animals. Statistical significance of relevant normal/dark reared post hoc comparisons is indicated.

Figure 2 presents western blot and densitometric analysis of α1- and α2-chimaerin proteins in the visual cortex of cats at the peak (5 weeks) and the nadir (20 weeks) of the critical period. For both isoforms, analysis of variance showed a significant interaction in cats (F  [1,8], p < .001), indicating that the effect of dark rearing depended on age. Both α-chimaerin proteins were significantly elevated (Tukey tests, p < .001) in dark reared compared to normal visual cortex at 5 weeks (α1: 2.6 fold; α2: 2.8 fold) and in normal compared to dark reared visual cortex at 20 weeks (α1: 1.6 fold; α2: 1.7 fold). In normal development, α1-chimaerin increased 5.6 fold from 5 to 20 weeks of age and α2-chimaerin increased 6.3 fold.

**Figure 3:**
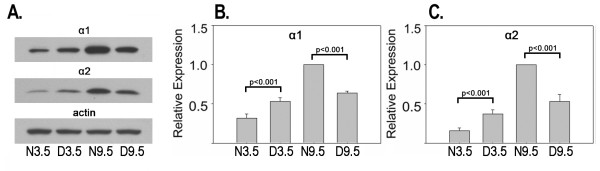
**Bidirectional regulation of α-chimaerin protein expression by age and dark rearing in mouse visual cortex.**  A. Western blots showing levels of α1- and α2-chimaerin and actin protein expression in mouse visual cortex. B and C. Mean levels of α-chimaerin isoform expression as determined by densitometry from three independent groups of mice. Means +/- S.E. are plotted. Data from each group were normalized against N9.5 animals. Statistical significance of relevant normal/dark reared post hoc comparisons is indicated.

**Figure 4:**
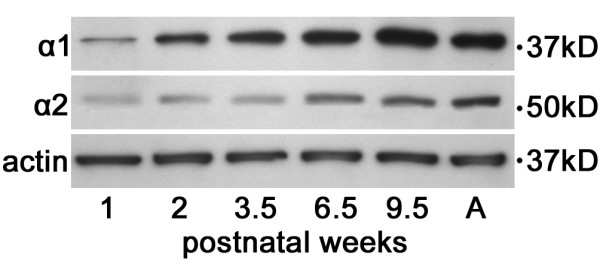
**Expression of α-chimaerin protein during normal development of mouse visual cortex**. Western blots showing expression of α1- and α2-chimaerin across normally reared postnatal development at 1, 2, 3.5, 6.5, 9.5 weeks and adult (18 weeks) in mouse visual cortex. The same blot was stripped and reprobed for both chimaerin isoforms and actin.

Figure 3 presents western blot and densitometric analysis from mouse visual cortex at the peak (3.5 weeks) and nadir (9.5 weeks) of the critical period. As in cats, for both proteins, analysis of variance showed a significant interaction in mice (F [1,8], p < .001), indicating that the effect of dark rearing depended on age. Both α-chimaerin isoforms were significantly elevated (Tukey tests, p < .001) in dark reared compared to normal visual cortex at 3.5 weeks (α1: 1.7 fold; α2: 2.3 fold) and in normal compared to dark reared visual cortex at 9.5 weeks (α1: 1.6 fold; α2: 1.9 fold). In normal development, α1-chimaerin increased 3.1 fold from 3.5 to 9.5 weeks of age and α2-chimaerin increased 6.1 fold. To provide a more complete description of visual cortical chimaerin expression across normal postnatal development, additional ages were analyzed in mice (figure 4). For both isoforms, expression was very low at one week of age, increased sharply from the time of eye opening (2 weeks) until the end of the critical period (9.5 weeks), and was stable thereafter until adulthood (18 weeks).

### Bidirectional Regulation of α-Chimaerin Protein Expression is not found in Frontal Cortex

**Figure 5:**
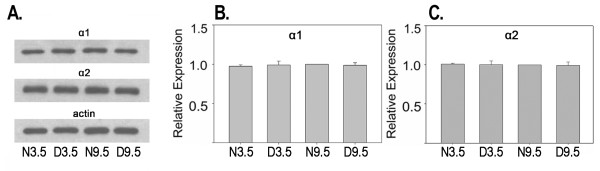
**Absence of bidirectional regulation of α-chimaerin protein expression by age and dark rearing in mouse frontal cortex. ** A. Western blots showing levels of α1-and α2-chimaerin and actin protein expression in mouse frontal cortex. B and C. Mean levels of α-chimaerin isoform expression as determined by densitometry from three independent groups of mice. Means +/- S.E. are plotted. Data from each group were normalized against N9.5 animals.

An important issue is whether the bidirectional regulation of α-chimaerin is specific to visual cortex and not a generalized phenomenon throughout the brain. If α-chimaerin is important for visual cortical plasticity, bidirectional regulation of its expression by age and dark rearing should be specific to or elevated in visual cortex. To answer this, we determined the effects of age and dark rearing on protein expression of both isoforms in mouse frontal cortex, as shown in figure 5. Levels of α-chimaerin isoform expression were similar in all rearing/age conditions. Densitometric analysis on three independent groups of mice indicated no statistically significant effects for either isoform in frontal cortex. Bidirectional regulation of α-chimaerin expression due to age and dark rearing was not a generalized effect throughout neocortex.

## Discussion

### Current Findings

The present ddPCR gene screening identified a novel candidate gene, α-chimaerin, as a candidate for a role in critical period neuroplasticity of the cat visual cortex. Western blot analysis confirmed that both α1- and α2-chimaerin proteins showed similar bidirectional regulation due to age and dark rearing in cat and mouse visual cortex and that this effect did not occur in frontal cortex. These combined results implicate both α-chimaerin isoforms as candidate molecules for neuronal plasticity during the visual cortical critical period. The pattern of bidirectional regulation shown by α-chimaerins was opposite to that shown by physiological plasticity [7,8]. Like Munc13-3 [10,11], α-chimaerin showed elevation in dark reared compared to normal animals at the peak of the normal critical period and elevation in normal compare to dark reared at the nadir. Genes that show this pattern could represent repressors of visual cortical plasticity. The present results provide the scientific basis for functional analyses of the effects of chimaerin gene mutation on visual cortical plasticity.

### Relation to previous gene screening studies

A growing number of gene screening studies have investigated postnatal age changes in gene expression or have manipulated neuronal activity levels to identify candidate neuronal plasticity genes. These approaches have yielded important information on a number of genes [19-28] that are responsive to visual input, developmentally regulated during the critical period, and/or involved in structural and functional plastic responses [see [29,30] for reviews]. All of these approaches confound changes in neuronal activity and changes in neuronal plasticity. The present bidirectional screen, based on the effects of age and dark rearing on physiologically assessed visual cortical plasticity, provides a promising alternative approach that more directly focuses on neuronal plasticity. Bidirectional regulation of gene expression is difficult to explain in terms of neuronal activity level. Dark rearing reduces the responsiveness of visual cortical neurons in both young and older cats [4,5]. Spontaneous activity is lower in young dark-reared animals and comparable in older dark-reared animals [5,31]. Therefore, the overall level of activity (spontaneous activity plus visual responses) in light-reared animals is higher than in dark-reared animals at all ages. If the expression of a gene reflected only neuronal activity level, it would be expected that dark rearing would have the same effect in both young and old animals and not show bidirectional regulation.

### α-Chimaerin in Neuronal Development and Plasticity

The two isoforms of α-chimaerin, α1 and α2, are alternatively spliced products of a single gene. Both isoforms are expressed in postnatal and adult brain; only α2 in embryonic brain [12]. Chimaerins are RhoGTPase-activating proteins (RhoGAPs) which activate or inactivate signaling pathways depending upon whether they are bound to GTP or GDP. The functions of chimaerins in the nervous system are just beginning to be understood [13-18]. Recently convergent evidence from three laboratories [14-16] implicated α2-chimaerin as an essential mediator of ephrinB3/EphA4-dependent motor circuit formation. Mutations of ephrinB3, EphA4 or α2-chimaerin produce a characteristic hopping rabbit like gait. This gait is due to a failure of α2-chimaerin mediated ephrin3 axonal guidance cues, resulting in bilateral corticospinal projections.

α2-Chimaerin has also been implicated in semaphorin induced growth cone collapse [13]. The interaction of chimaerins with semaphorin raises another possible mechanism by which age/dark rearing and their regulation of α2-chimaerin could regulate neuronal plasticity. Semaphorins are also critical factors in angiogenesis [32]. In addition to its well documented effects on visual cortical neuronal function, dark rearing also affects astrocytes [33] and vasculature [34]. Thus, the entire neuronal-glial-vasculature network could be involved in the contribution of chimaerin to neuronal plasticity. This concept is central to the brain/cognitive reserve hypothesis which has been proposed to explain neuronal plasticity induced by pathology or trauma [35,36].

α1-Chimaerin has been implicated in regulation of in dendrites and spines. Increased expression of α1-chimaerin expression results in pruning and suppression of α1-chimaerin in expansion of dendritic spines and branches [17,18]. α1-Chimaerin is present in dendrites and spines, where it binds to the NMDA receptor NR2A subunit and the ability of α1-chimaerin to modulate dendritic spines is dependent upon an interaction with the NMDA receptor [18]. α-Chimaerins are promising newly identified players in axonal development and synaptic plasticity. The present results extend this evidence by implicating α- chimaerins as candidate molecules for a role in postnatal critical period plasticity of visual cortex.

## Conclusions

The present results identify α-chimaerin as a candidate molecule for a role in the postnatal critical period of visual cortical plasticity. Electrophysiological results had shown that dark rearing slows the entire time course of this critical period, such that at 5 weeks of age, normal cats are more plastic than dark reared cats, whereas at 20 weeks, dark reared cats are more plastic. ddPCR, northern blotting and sequencing identified a novel gene, α1-chimaerin, as a candidate visual cortex critical period plasticity gene that showed such bidirectional regulation of expression due to age and dark rearing. Western blotting extended the evidence for bidirectional regulation of expression of both visual cortical α-chimaerin isoforms to protein in cats and mice. α1- and α2-Chimaerin were elevated in dark reared compared to normal visual cortex at the peak of the normal critical period and in normal compared to dark reared visual cortex at the nadir of the normal critical period. Genes that show this pattern could represent repressors of visual cortical plasticity. The present results provide the scientific basis for functional analyses of the effects of chimaerin gene mutation on visual cortical plasticity.

## Methods

### Animals

Cats were used for differential display PCR, cloning, sequencing, northern and western blotting. Cats were reared in a normal 12 hour light/dark (N) cycle or in complete darkness from birth to 5 or 20 weeks of age (D). For each of these four rearing/age conditions (N5, D5, N20, D20), three animals were used (12 cats in total). Fresh brain regions (visual cortex [all of Area 17 and possibly a small part of 18], frontal cortex [the anterior quarter of the cerebral hemisphere], and cerebellum [entire structure]) were dissected, immediately frozen in liquid nitrogen, and stored at -80°C until used for extraction of total RNA and protein.

Mice were used for western blot analysis of α-chimaerin isoform expression. They were reared in a normal 12 hour light/dark cycle or in complete darkness from birth to 3.5 or 9.5 weeks of age. The ages were based on published studies of the time course of the critical period in mouse visual cortex [37-39]. Three independent samples of mice including all four ages and rearing conditions (N3.5, D3.5, N9.5, D9.5) were collected. Within each sample three animals were pooled for each rearing/age condition (36 mice in total) to provide sufficient tissue and reduce individual variability. To provide a complete description of normal postnatal development of chimaerin expression in visual cortex, additional mice were reared in a normally lit environment until 1, 2, 6.5 weeks, and adult (18 weeks). Three animals were pooled for each age sample as above (total 12 additional mice). Visual cortex (monocular and binocular regions), frontal cortex and in several normal 9.5 week mice cerebellum were dissected, immediately frozen in liquid nitrogen, and stored at -80°C until used for western blots. Cats and mice were killed by an overdose of sodium pentobarbital prior to tissue dissection (75 mg/kg, intraperitoneal injection). All procedures in cats and mice conformed to the guidelines of the National Institutes of Health and were approved by the Institutional Animal Care and Use Committee.

### Differential Display PCR (ddPCR)

RNA was extracted from cat visual cortical samples and its amount and integrity determined as described previously [40]. DNA contamination was removed from the RNA (MessageClean, GenHunter Corp., Nashville, TN) and cDNA was synthesized (SMART, Clontech, Palo Alto, CA or RNAimage, GenHunter Corp.). PCR on cDNA from each RNA sample was done using the appropriate oligo-dT-X (oligo-dT-C; dT-G; dT-A) primer and 80 arbitrary primers as provided in a commercially available kit (RNAimage, GenHunter Corp.). The ddPCR products showing bidirectional expression due to age and dark rearing were amplified in a PCR reaction using the same primers as in the initial reaction, then cloned (PCR-TRAP, GenHunter) for use in sequencing and as probes for northern blots. The resultant sequences were run against gene and expressed sequence tag databases to determine similarity to known genes. 5'RACE PCR and cloning (SMART, Clontech) were used to generate additional sequence for the ddPCR fragment.

### Northern Blot Analysis

The cat ddPCR fragment (153 bp) obtained from the screen was used as the probe for northern blotting. Total RNA was extracted from the tissue samples as described above. Filters with lanes from all experimental conditions were hybridized (stringency: 0.1x sodium chloride-sodium citrate buffer, 0.1x sodium dodecyl sulphate (SDS), 42°C) using these probes according to our standard procedures [40]. The filters were stripped and re-hybridized with a probe to glyceraldehyde-3-phosphate dehydrogenase (GAPDH) to correct for loading errors (American Type Culture Collection, ATCC 57090). The relative intensity of signals in northern blots was determined by densitometric scanning, corrected against GAPDH.

### Western Blot Analysis

Antibodies against α1- and α2-chimaerin were developed in collaboration with Quality Controlled Biochemicals (Hopkinton, MA.) The antibody to the α1 isoform was developed against amino acids 9-29 and the antibody to α2 against amino acids 151-173. Both antibodies were affinity purified. Homogenates of visual and frontal cortex were prepared in the presence of a standard protease inhibitor cocktail (Sigma-Aldrich Co., St. Louis, MO). Centrifugation was done to yield a crude synaptosomal fraction and equal amounts of protein were added to each well of a 4-15% polyacrylamide SDS gel (Bio-Rad Laboratories, Inc., Hercules, CA), separated by electrophoresis, and then electrophoretically transferred to a polyvinylidene fluoride membrane using a Transblot cell (Bio-Rad Laboratories, Inc., Hercules, CA). Nonspecific binding sites were blocked with 5% non-fat milk and blots were then incubated with the primary antibody (rabbit polyclonal, diluted 1:1000). Blots were extensively washed and incubated with appropriate horseradish peroxidase conjugated secondary antibodies at room temperature for 2 hr. Specific protein bands were visualized by enhanced chemiluminescence (ECL) detection reagents (Amersham Biosciences, Piscataway, NJ). The blots processed for ECL were then stripped and reprobed with the other chimaerin antibody, then stripped again and reprobed with a mouse monoclonal antibody to β-actin (Sigma-Aldrich Co., St. Louis, MO) to control for loading errors. The second and third immunostaining were carried out as described above.

Exposed films from western blots were quantified by computerized densitometric analysis. High resolution TIF images of each film were obtained with an AlphaImager EP, MultiImage I. Protein expression levels for each individual antibody were calculated as an average pixel density by AlphaView software (version 2.0.0.9) from AlphaInnotech. All densitometric measures for α-chimaerin protein were corrected against β-actin. Statistical analysis included two way ANOVA and Tukey post hoc comparisons.

## Competing interests

The authors declare that they have no competing interests.

## Authors' contributions

CBY performed ddPCR, cloning, sequencing, antibody development, northern and western blotting, and assisted in data analysis and manuscript preparation. YTZ performed bioinformatic analyses and participated in sequencing and antibody development. PJK carried out animal rearing, tissue dissection, data analysis and assisted in drafting the manuscript. GDM conceived of the study, directed its design and execution, conducted data analysis and drafted the manuscript. All authors read and approved the final manuscript.
